# Algorithmic Mechanisms for Reliable Crowdsourcing Computation under Collusion

**DOI:** 10.1371/journal.pone.0116520

**Published:** 2015-03-20

**Authors:** Antonio Fernández Anta, Chryssis Georgiou, Miguel A. Mosteiro, Daniel Pareja

**Affiliations:** 1 IMDEA Networks Institute, Madrid, Spain; 2 Dept. of Computer Science, University of Cyprus, Nicosia, Cyprus; 3 Dept. of Computer Science, Kean University, Union, New Jersey, United States of America; Tianjin University of Technology, CHINA

## Abstract

We consider a computing system where a master processor assigns a task for execution to worker processors that may collude. We model the workers’ decision of whether to comply (compute the task) or not (return a bogus result to save the computation cost) as a game among workers. That is, we assume that workers are rational in a game-theoretic sense. We identify analytically the parameter conditions for a unique Nash Equilibrium where the master obtains the correct result. We also evaluate experimentally mixed equilibria aiming to attain better reliability-profit trade-offs. For a wide range of parameter values that may be used in practice, our simulations show that, in fact, both master and workers are better off using a pure equilibrium where no worker cheats, even under collusion, and even for colluding behaviors that involve deviating from the game.

## Introduction

### Motivation and prior work

The need for high-performance computing, the partial availability of resources in personal computers (such as CPU and GPU), and the wide access to the Internet, have led to the development of Internet-based computing. Internet-based computing is mostly embraced by the scientific community in the form of volunteer computing. That is, a system where participants contribute their idle computing resources to solve scientific problems. Among the most popular volunteering projects is SETI@home [1] running on the BOINC [2] platform. Computing platforms where users contribute for profit also exist, for example, Amazon’s Mechanical Turk [3]. Unfortunately, although the potential of such systems is great, the use of Internet-based computing is constrained by the untrustworthy nature of the platform’s components [2, 4].

In Internet-based Master-Worker task computing systems a *master* process sends tasks, across the Internet, to *worker* processes. Workers are expected to execute and report back the result. However, these workers are not trustworthy, hence, they might report incorrect results [2, 5, 6]. Prior work has considered different approaches in tackling this shortcoming. A “classical” Distributed Computing approach is to model malfunctioning (hardware or software errors) or cheating (intentional wrongdoing) as *malice*. That is, it is assumed the presence of some workers that wish to hamper the computation returning always an incorrect result. On the other hand, non-malicious workers are modeled as *altruism*. That is, it is assumed the presence of other workers who always return the correct result. Under this classical model, malice-tolerant protocols have been considered [7–9] where the master takes the result returned by the majority of workers. A Game-theoretic approach to deal with untrustworthiness is to assume that workers are *rational* [4, 10, 11]. That is, based on the strategy that best serves its self-interest, a worker decides whether to compute and return the correct result or return a bogus result. Under this game-theoretic model, incentive-based algorithmic mechanisms have been devised [12–14]. These mechanisms employ reward/punishment schemes to “enforce” rational workers to act correctly. More recent works [15, 16] have combined these two approaches and devised mechanisms assuming the co-existence of altruistic, malicious and rational workers. For a discussion on the connection between Game Theory and (distributed) computing we refer the reader to the book by Nisan et al. [17] and the survey by Abraham et al. [18].

With respect to collusion, in [19, 20] workers are assumed to have a predefined behavior (they are always faulty or always correct). In both papers, the goal is to identify colluders statistically, which requires multiple rounds of worker computation. Rather than focusing on colluder detection, in the present paper, we design mechanisms that yield the correct task computation, *despite* collusions. Furthermore, in our work each worker computes a task at most once. The benefit of one-round per task mechanisms is partially supported by the work of Kondo et al. [21]. In such work it was demonstrated experimentally that, in master-worker computations, tasks may take much more than one day of CPU time to complete.

In a work directly related to the present paper [14], master-worker computations with rational colluding workers are also studied. In their model, the master can audit the results returned by rational workers with a tunable probability. Bounds on the audit probability that guarantee that workers have incentives to be honest are shown. The study is carried out for three scenarios: redundant allocation of tasks with and without collusion, and single-worker allocation. They conclude that, for their model, single-worker allocation is a cost-effective mechanism, specially in presence of collusion. The form of collusion considered is close to ours: within the same group, either all processors cheat or are honest. However, our analysis and simulations provide a much deeper understanding of the impact of collusions on the master-worker problem. Furthermore, our work considers a richer payoff model and probabilistic cheating, yielding interesting tradeoffs between the utility of the master and the probability of obtaining an incorrect result.

To the best of our knowledge, with the exception of the works discussed above, workers have been always assumed to interact only with the master. That is, no explicit worker collusion that models interaction among workers is considered. (In some works, it is assumed that cheating workers would return the same incorrect answer, without them interacting with each other. This is an implicit and weak form of collusion assumed as a worst case for a voting-based mechanism, as workers returning different incorrect answers would less likely reach a majority.) However, one cannot exclude the possibility of collusion, for example, as in Sybil attacks [22]. In this paper, we study the impact of collusion and the tradeoffs involved in Internet-based Master-Worker task computing, under the assumption that workers are rational in a game-theoretic sense.

Other related work in the area of cloud service and cloud computing includes [23, 24]. Although the overall setting and methodologies considered are different from ours, their objective is similar to ours: increase the trustworthiness of the system.

### Contributions

Our main contributions are as follows:

**1.** We model the Master-Worker Internet-based task computation as a game among rational workers under collusion (cf. Section 2). Each worker chooses whether to be *honest* (i.e., compute and return the correct task result) or a *cheater* (i.e., fabricate a bogus result and return it to the master) based on which of the two strategies increases its *utility* (benefit). In addition, we consider worker *collusion*: collections of workers form groups. Within each group, workers decide on a common strategy that would provide greater expected benefit. Neither the master nor other workers outside of a colluding group are aware of the collusion. The model is enhanced with a collection of realistic payoff parameters to quantify the benefit of the master and the workers. These parameters can either be fixed because they are system parameters or be chosen by the master.

**2.** Under this model, we design algorithmic mechanisms that provide the necessary incentives for the workers to truthfully compute and report the correct result, despite collusion. The objective of the design is twofold: maximize the probability of obtaining the correct result (reliability) and maximize the master utility (maximizing profit or minimizing cost if profit is not possible). In short, the mechanism works as follows (cf. Section 4): The master assigns the task to *n* workers. Each worker *i* cheats with probability $p_{C}^{\left(i\right)}$ and the master verifies the answers with some probability *p*_*V*_ (verification comes at a cost–see Section 2). If the master verifies, it rewards the honest workers and penalizes the cheaters. If the master does not verify, it accepts the answer returned by the majority of workers and rewards these workers only. However, it does not penalize the workers in the minority, given that the majority may be actually cheating. Workers may collude in which case the workers belonging to the same group decide homogeneously: either all cheat or all are honest (for more details on collusion see Section 2).

**3.** The workers’ decision of whether to cheat or not is modeled as a game. As typical in algorithmic mechanism design [10, 25], the master induces a *Nash Equilibrium* (NE) [26]. When this NE is followed by the workers, the outcome has the desired properties (reliability and utility). We analyze the game and identify the conditions under which unique NE is achieved (cf Section 3). The reason for uniqueness is to force all workers to the same strategy (this is similar to *strong implementation* in Mechanism Design [27]). Each unique NE results in a different benefit for the master and a different probability of accepting an incorrect result. Thus, the master can choose the game conditions for the unique NE that best fits its goals.

**4.** To demonstrate the practicality of the analysis, we design mechanisms for two specific realistic scenarios. These scenarios reflect, in their fundamental elements, (a) a system of volunteer computing (e.g., SETI), and (b) a company that buys computing cycles from participant computers and sells them to its customers in the form of a task-computation service (e.g., Amazon’s Mechanical Turk). The analysis provided for these scenarios comprises implicitly a mechanism to decide how to carry out the computation. Our analysis reveals interesting tradeoffs between reliability and utility (see Section 4). A general conclusion drawn from the analysis with respect to worker collusion is that, in order to guarantee a unique equilibrium for any parameter values, all groups, colluding or singletons, must decide whether to cheat or not deterministically.

**5.** We carry out extensive simulations of our mechanisms for the contractor scenario in an attempt to better understand the impact of worker collusion (cf Section 5). Specifically our simulations are designed to investigate whether considering *mixed* equilibria yields better tradeoffs for the master and/or the workers, and whether the workers would benefit more if they did not follow the NE induced by the master (i.e., deviate from the game). For a wide range of parameter values (that may be used in practice), our simulations show that all parties, the master and the workers, are better-off using a pure equilibrium where no worker cheats, *even under collusion*, and even for colluding behaviors that involve deviating from the game.

## Model and Definitions

### Framework

*Setting:* We consider a distributed system consisting of a master processor that assigns a computational task to a set of workers to compute and return the result. The tasks considered in this work are assumed to have a unique solution. Although such limitation reduces the scope of application of the mechanisms presented [28], there are plenty of computations where the correct solution is unique, e.g., any mathematical function.

*Master verification and worker rationality:* It is assumed that the master has the possibility of *verifying* whether the value returned by a worker is the correct result of the task. It is also assumed that verifying an answer is computationally easier than computing the task [29] (e.g., numerical computations), but the correct result of the computation is not obtained if the verification fails (e.g., when all workers cheat). (Alternatively, one might assume that the master verifies by simply performing the task and checking the answers of the workers. Our analysis can easily be modified to accommodate this different model.) As in [12–14], workers are assumed to be *rational* and seek to maximize their benefit, i.e., they are not destructively malicious. We note that this assumption can conceptually be justified by the work of Shneidman and Parkes [11] where they reason on the connection of rational players (of Algorithmic Mechanism Design) and workers in realistic P2P systems. Furthermore, we do not consider unintentional errors produced by hardware or software problems. As already mentioned, the master verifies with probability *p*_*V*_, and the each worker *i* cheats with probability $p_{C}^{\left(i\right)}$.

*Collusion:* We consider a form of collusion that covers realistic types such as Sybil attacks [22]). Within any given colluding group workers act homogeneously, i.e., either all choose to cheat, or all choose to be honest. The decision is perhaps randomized tossing a unique coin. In the case that a colluding group chooses to be honest, then only one of the workers computes the task (saving computing cost). In the case that that a colluding group chooses to cheat, then they simply agree on a bogus result. In either case they return the agreed result to the master. In addition, we assume that all “cheating groups” return the same incorrect answer if there is more than one. Both assumptions, homogeneous behavior within groups and unique incorrect answer, are adversarial. Since the master accepts the majority, this colluding strategy maximizes the chances of cheating the master. Being this the worst case (see also [9]), it subsumes models where cheaters do not necessarily return the same answer. We assume that, if a worker does not compute the task, the probability of guessing the correct answer is negligible. We also assume that the overall number of colluders is not a majority. The assumption comes naturally from the fact that the set of workers is a choice of the master, who can select arbitrarily from a large population making the likelihood of choosing many related workers low, specially for a one-shot computation. With respect to the master, we assume that it is not aware of which workers are colluding, although it is aware that collusion may occur.

### Game definition

We now define formally a game “played” by the workers in a game theoretic sense. We summarize the notation in Table 1 for easy reference. We assume that the master hires an odd number of workers *n* ≥ 3 to be able to apply a voting strategy and to avoid ties. In order to model collusion among workers, we view the set of workers as a set of non-empty subsets *W* = {*W*_1_, …, *W*_ℓ_} such that $\sum_{i=1}^{\ell }|W_{i}|=n$ and *W*_*i*_ ∩ *W*_*j*_ = ∅ for all *i* ≠ *j*, 1 ≤ *i*, *j* ≤ *ℓ*. We refer to each of these subsets as a *group of workers* or a *group* for short. We also refer to groups as *players*. Workers acting individually are modeled as a group of size one. It is assumed that the size of each group is known only to the members of the group. Other than that, we assume that workers have complete information on the algorithm and the parameters involved.

**Table 1:** Game notation.

*W* = {*W*_1_, …, *W*_ℓ_}	set of worker groups
$S_{i}=\{C,\bar{C}\}$	set of pure strategies (cheat/not-cheat) available to group *W*_*i*_
*s*	strategy profile (a mapping from players to pure strategies)
*s*_*i*_	strategy used by group *W*_*i*_ in the strategy profile *s*
*s*_−*i*_	strategy used by each player but *W*_*i*_ in the strategy profile *s*
$w_{s}^{\left(i\right)}$	payoff of group *W*_*i*_ for the strategy profile *s*
*σ*	mixed strategy profile (a mapping from players to prob. distrib. over pure strategies)
*σ*_*i*_	probability distribution over pure strategies used by group *W*_*i*_ in *σ*
*σ*_−*i*_	probability distribution over pure strategies used by each player but *W*_*i*_ in *σ*
$p_{s_{i}}^{\left(i\right)}$	probability that group *W*_*i*_ uses strategy *s*_*i*_
*supp*(*σ*_*i*_)	set of strategies of group *W*_*i*_ with probability > 0 (called support) in *σ*
*U*_*i*_(*s*_*i*_, *σ*_−*i*_)	expected utility of group *W*_*i*_ with mixed strategy profile *σ*

For succinctness, we express a strategy profile as a collection of individual strategy choices together with collective strategy choices. For instance, *s*_*i*_ = *C*, *R*_−*i*_, *F*_−*i*_, *T*_−*i*_ stands for a strategy profile *s* where group *W*_*i*_ chooses strategy *C* (to cheat), a set *R*_−*i*_ of groups (where group *W*_*i*_ is not included) randomize their strategy choice with probability *p*_*C*_ ∈ (0, 1), a set *F*_−*i*_ of groups deterministically choose strategy *C*, and a set *T*_−*i*_ of groups deterministically choose strategy $\bar{C}$ (to be honest). We require that, for each group *W*_*i*_, $p_{C}^{\left(i\right)}=1-p_{\bar{C}}^{\left(i\right)}$. Whenever all groups use the same probability, we drop the superindex (*i*) for clarity. Also, whenever the strategy is clear from context, we refer to the expected utility of group *W*_*i*_ as *U*_*i*_.

### Equilibrium definition

We define now the conditions for the equilibrium. In the context of collusion, the probability distributions are not independent among members of a group. Furthermore, the formulation of equilibrium conditions among individual workers would violate the very definition of equilibrium since the choice of a worker does change the choices of other workers. Instead, equilibrium conditions are formulated among groups. Of course, the computation of an equilibrium might not be possible since the size of the groups is unknown. But, finding appropriate conditions so that the unique equilibrium is the same independently of that size, the problem may be solved. As we will see in the analysis, knowing some bound (e.g. the trivial one) on the size of the smallest and/or largest group is enough. Moreover, as we will see in simulations, deviating from this expected behavior is against workers’ interest in practice. It is important to notice that although the majority is evaluated in terms of number of single answers, this fact has no impact on correctness of the equilibrium formulation.

We recall [30] that, for any finite game, a mixed strategy profile σ* is a *mixed-strategy Nash equilibrium* (MSNE) if, and only if, for each player *π*,

$$U_{\pi }\left(s_{\pi },\sigma_{-\pi }^{*}\right)=U_{\pi }\left(s_{\pi }^{'},\sigma_{-\pi }^{*}\right),\forall s_{\pi },s_{\pi }^{'}\in supp\left(\sigma_{\pi }^{*}\right),$$ (1)

$$U_{\pi }\left(s_{\pi },\sigma_{-\pi }^{*}\right)\geq U_{\pi }\left(s_{\pi }^{'},\sigma_{-\pi }^{*}\right),\forall s_{\pi },s_{\pi }^{'}\;:\;s_{\pi }\in supp\left(\sigma_{\pi }^{*}\right),s_{\pi }^{'}\notin supp\left(\sigma_{\pi }^{*}\right).$$ (2)

Where $\sigma_{\pi }^{*}$ is the probability distribution over pure strategies used by *π* in σ*, $\sigma_{-\pi }^{*}$ is the probability distribution over pure strategies used by each player but *π* in σ*, $supp(\sigma_{\pi }^{*})$ is the set of strategies of *π* with probability > 0 (called support) in σ*, and $U_{\pi }(s_{\pi },\sigma_{-\pi }^{*})$ is the expected utility of *π* in σ*.

In words, given a MSNE with mixed-strategy profile σ*, for each player *π*, the expected utility, assuming that all other players do not change their choice, is the same for each pure strategy that the player can choose with positive probability in σ*, and it is not less than the expected utility of any pure strategy with probability zero of being chosen in σ*. A *fully* MSNE is an equilibrium with mixed strategy profile *σ* where, for each player *π*, *supp*(*σ*_*π*_) = *S*_*π*_, where *S*_*π*_ is the whole set of pure strategies available to *π*.

### Payoffs definition

We detail in Table 2 the workers’ payoffs. We also include master payoffs to evaluate its utility. All the parameters in this table are non-negative.

**Table 2:** Master’s and Workers’ Payoffs.

*WP*_*C*_	worker’s punishment for being caught cheating
*WC*_*T*_	group’s cost for computing the task
*WB*_*A*_	worker’s benefit from master’s acceptance
*MP*_*W*_	master’s punishment for accepting a wrong answer
*MC*_*A*_	master’s cost for accepting the worker’s answer
*MC*_*V*_	master’s cost for verifying worker’s answers
*MB*_*R*_	master’s benefit from accepting the right answer

Notice that we split the reward to a worker into *WB*_*A*_ and *MC*_*A*_, to model the fact that the cost of the master might be different than the benefit of a worker. In fact, in some models they may be completely unrelated. Among the parameters involved, we assume that the master has the freedom of choosing the cheater penalty *WP*_*C*_ and the worker’s reward *WB*_*A*_. By tuning these parameters and choosing *n*, the master achieves the desired trade-off between reliability and utility. Recall that the master does not know the composition of groups (if there is any). Hence, benefits and punishments are applied individually to each worker, except for the cost for computing the task *WC*_*T*_ which is shared among all workers in the same group. Sharing the cost of computing while being paid/punished individually may provide incentive to collude, but it models the real-world situation where collusion is secret.

## Equilibria Analysis

From Eqs. (1) and (2), we obtain conditions on payoffs and probabilities to attain a unique NE. A similar analysis was presented in [13] for various games and reward models, but for a model *without collusion*. The analysis presented here can be also applied to those models and games where collusion may appear. We consider a game where the master assigns a computing task to *n* workers that “play” (in a game-theoretic sense) among them. Intuitively, it is to the workers’ advantage to be in the majority in case the master does not verify and the majority is rewarded. Given that workers know the existence of the other workers, including collusions in the analysis is in order.

For clarity, we partition the set of groups as {*F*, *T*, *R*}, where *F* ∪ *T* ∪ *R* = *W*. *F* is the set of groups that choose to cheat as a pure strategy, that is, $F=\{W_{i}|W_{i}\in W\wedge p_{C}^{\left(i\right)}=1\}$. *T* is the set of groups that choose not to cheat as a pure strategy, that is, $T=\{W_{i}|W_{i}\in W\wedge p_{C}^{\left(i\right)}=0\}$. *R* is the set of groups that randomize their choice, that is, $R=\{W_{i}|W_{i}\in W\wedge p_{C}^{\left(i\right)}\in (0,1)\}$. Let *F*_−*i*_ = *F* \ {*W*_*i*_}, *T*_−*i*_ = *T* \ {*W*_*i*_}, and *R*_−*i*_ = *R* \ {*W*_*i*_}. Let Γ_−*i*_ be the set of partitions in two subsets (*R*_*F*_, *R*_*T*_) of *R*_−*i*_, i.e., Γ_−*i*_ = {(*R*_*F*_, *R*_*T*_)∣*R*_*F*_ ∩ *R*_*T*_ = ∅ ∧ *R*_*F*_ ∪ *R*_*T*_ = *R*_−*i*_}. Let $\text{E}[w_{s}^{\left(i\right)}]$ be the expected payoff of group *W*_*i*_ for the strategy profile *s*, taking the expectation over the choice of the master of verifying or not. Then, for each group *W*_*i*_ ∈ *W* and for each strategy profile *s*_−*i*_ = *R*_−*i*_, *F*_−*i*_, *T*_−*i*_, we have that $U_{i}(s_{-i},s_{i}=C)=\sum_{(R_{F},R_{T})\in \Gamma_{-i}}\prod_{W_{f}\in R_{F}}p_{C}^{\left(f\right)}\prod_{W_{t}\in R_{T}}(1-p_{C}^{\left(t\right)})\text{E}[w_{s^{\prime }}^{\left(i\right)}]$, where *s*′ is the strategy profile *F*_−*i*_ ∪ *R*_*F*_, *T*_−*i*_ ∪ *R*_*T*_, *s*_*i*_ = *C*; and that $U_{i}(s_{-i},s_{i}=\bar{C})=\sum_{(R_{F},R_{T})\in \Gamma_{-i}}\prod_{W_{f}\in R_{F}}p_{C}^{\left(f\right)}\prod_{W_{t}\in R_{T}}(1-p_{C}^{\left(t\right)})\text{E}[w_{s^{\prime \prime }}^{\left(i\right)}]$, where *s*″ is the strategy profile $F_{-i}\cup R_{F},T_{-i}\cup R_{T},s_{i}=\bar{C}$.

In order to find conditions for a desired equilibrium, we study what we call the *utility differential* of a worker, i.e. the difference on the expected utility of a worker if its group chooses to cheat with respect to the case when the group chooses to be honest. That is, $\Delta U_{i}(s)=U_{i}(s_{-i},s_{i}=C)-U_{i}(s_{-i},s_{i}=\bar{C})$.

For clarity, define *N*_*F*−*i*_ = ∑_*S* ∈ *F*_−*i*_∪*R*_*F*__ ∣*S*∣ and *N*_*T*−*i*_ = ∑_*S* ∈ *T*_−*i*_∪*R*_*T*__ ∣*S*∣, i.e. the number of cheaters and honest workers respectively except for those in group *W*_*i*_. We also define what we call the *payoff differential* as the difference on the expected payoff of a worker, the expectation taken over the choice of the master, if its group chooses to cheat with respect to the case when the group chooses to be honest. Furthermore, we denote the payoff differential depending on whether the size of the group has an impact on what is the majority outcome. More precisely, for each partition (*R*_*F*_, *R*_*T*_) ∈ Γ_*i*_, let $\Delta w_{C}^{\left(i\right)}=\text{E}[w_{s_{i}=C}^{\left(i\right)}]-\text{E}[w_{s_{i}=\bar{C}}^{\left(i\right)}]$, when *N*_*F*−*i*_ − *N*_*T*−*i*_ > ∣*W*_*i*_∣, $\Delta w_{C}^{\left(i\right)}=E[w_{s_{i}=C}^{\left(i\right)}]-E[w_{s_{i}=\bar{C}}^{\left(i\right)}]$, when *N*_*T*−*i*_ − *N*_*F*−*i*_ > ∣*W*_*i*_∣, and $\Delta w_{X}^{\left(i\right)}=\text{E}[w_{s_{i}=C}^{\left(i\right)}]-\text{E}[w_{s_{i}=\bar{C}}^{\left(i\right)}]$, when ∣*N*_*F*−*i*_ − *N*_*T*−*i*_∣ < ∣*W*_*i*_∣.

Given that the payoff depends only on the outcome majority, replacing this notation in the utility differential, we have that Δ*U*_*i*_(*s*) is

$$\begin{array}{cc} & \Delta w_{C}^{\left(i\right)}\;\sum_{\begin{array}{c} \left(R_{F},R_{T}\right)\in \Gamma_{-i}\\ N_{F-i}-N_{T-i}>|W_{i}| \end{array}}\prod_{W_{f}\in R_{F}}p_{C}^{\left(f\right)}\prod_{W_{t}\in R_{T}}\left(1-p_{C}^{\left(t\right)}\right)+\\ & \Delta w_{X}^{\left(i\right)}\;\sum_{\begin{array}{c} \left(R_{F},R_{T}\right)\in \Gamma_{-i}\\ |N_{F-i}-N_{T-i}|<|W_{i}| \end{array}}\prod_{W_{f}\in R_{F}}p_{C}^{\left(f\right)}\prod_{W_{t}\in R_{T}}\left(1-p_{C}^{\left(t\right)}\right)+\\ & \Delta w_{\bar{C}}^{\left(i\right)}\;\sum_{\begin{array}{c} \left(R_{F},R_{T}\right)\in \Gamma_{-i}\\ N_{T-i}-N_{F-i}>|W_{i}| \end{array}}\prod_{W_{f}\in R_{F}}p_{C}^{\left(f\right)}\prod_{W_{t}\in R_{T}}\left(1-p_{C}^{\left(t\right)}\right). \end{array}$$ (3)

Restating Eqs. (1) or (2) in terms of Eq. (3), the equilibrium conditions are, for each group that does not choose a pure strategy, the differential utility must be zero (∀*i* ∈ *R*, Δ*U*_*i*_(*s*) = 0); for each group that chooses to cheat as a pure strategy, the differential utility must not be negative (∀*i* ∈ *F*, Δ*U*_*i*_(*s*) ≥ 0); and for each group that chooses to be honest as a pure strategy, the differential utility must not be positive (∀*i* ∈ *T*, Δ*U*_*i*_(*s*) ≤ 0).

The following lemma, which is crucially used in the rest of our analysis, shows that, if there is a given total order among the payoff differentials defined, in order to attain a unique equilibrium all groups must decide deterministically. The proof is based on an algebraic argument.

**Lemma 1**. *Given a game as defined, if* $\Delta w_{C}^{\left(i\right)}\geq \Delta w_{X}^{\left(i\right)}\geq \Delta w_{\bar{C}}^{\left(i\right)}$ *for every group* *W*_*i*_ ∈ *W*, *then there is no unique equilibrium where* *R* ≠ ∅ *(i.e, all groups decide deterministically)*.

*Proof*. For the sake of contradiction, assume there is a unique equilibrium *σ* for which *R* ≠ ∅ and $\Delta w_{C}^{\left(i\right)}\geq \Delta w_{X}^{\left(i\right)}\geq \Delta w_{\bar{C}}^{\left(i\right)}$ for every group *W*_*i*_ ∈ *W*. Then, for every group *W*_*i*_ ∈ *R*, Δ*U*_*i*_(*s*) = 0 must be solvable. If $\Delta w_{C}^{\left(i\right)}\geq 0$, for all *W*_*i*_ ∈ *R*, there would be also an equilibrium where all groups in *R* choose to cheat and *σ* would not be unique, which is a contradiction. Consider now the case where there exists some *W*_*i*_ ∈ *R* such that $\Delta w_{C}^{\left(i\right)}<0$. Then, it must hold that ∣*R*∣ > 1, otherwise Δ*U*_*i*_ = 0 is false for *W*_*i*_. Given that ∣*R*∣ > 1, the probabilities given by the summations in Equation (3) for *W*_*i*_ are all strictly bigger than zero. Therefore, given that Δ*U*_*i*_ = 0 must be solvable, at least one of $\Delta w_{X}^{\left(i\right)}>0$ and $\Delta w_{\bar{C}}^{\left(i\right)}>0$ must hold, which is also a contradiction with the assumption that $\Delta w_{C}^{\left(i\right)}\geq \Delta w_{X}^{\left(i\right)}\geq \Delta w_{\bar{C}}^{\left(i\right)}$.

Replacing appropriately the payoffs detailed in Table 2, we obtain for any group *W*_*i*_ ∈ *W*

$$\Delta w_{C}^{\left(i\right)}=-p_{V}|W_{i}|\left({\mathrm{WP}}_{C}+2{\mathrm{WB}}_{A}\right)+|W_{i}|{\mathrm{WB}}_{A}+{\mathrm{WC}}_{T},$$ (4)

$$\Delta w_{X}^{\left(i\right)}=-p_{V}|W_{i}|\left({\mathrm{WP}}_{C}+{\mathrm{WB}}_{A}\right)+{\mathrm{WC}}_{T},$$ (5)

$$\Delta w_{\bar{C}}^{\left(i\right)}=-p_{V}|W_{i}|{\mathrm{WP}}_{C}-|W_{i}|{\mathrm{WB}}_{A}+{\mathrm{WC}}_{T}.$$ (6)

In the equations above, it holds that $\Delta w_{C}^{\left(i\right)}\geq \Delta w_{X}^{\left(i\right)}\geq \Delta w_{\bar{C}}^{\left(i\right)}$ for all *W*_*i*_ ∈ *W*. Then, by Lemma 1, there is no unique equilibrium where *R* ≠ ∅. Regarding equilibria where *R* = ∅, unless the task assigned has a binary output (i.e. the answer can be negated), a unique equilibrium where all groups choose to cheat is not useful to the master. Then, we set up *p*_*V*_ so that $\Delta w_{C}^{\left(i\right)}<0$, $\Delta w_{X}^{\left(i\right)}<0$ and $\Delta w_{\bar{C}}^{\left(i\right)}<0$ for all *W*_*i*_ ∈ *W* so that Δ*U*_*i*_ ≥ 0 has no solution and no group can choose to cheat as a pure strategy. Thus, the only equilibrium is for all the groups to choose to be honest, which solves Δ*U*_*i*_ ≤ 0. Therefore, $p_{C}^{\left(i\right)}=0$, ∀*W*_*i*_ ∈ *W* and, hence, the probability that the master accepts a wrong answer is **P**_*wrong*_ = 0.

To make $\Delta w_{C}^{\left(i\right)}<0$ we need *p*_*V*_ > (∣*W*_*i*_∣*WB*_*A*_ + *WC*_*T*_)/(∣*W*_*i*_∣(*WP*_*C*_ + 2*WB*_*A*_)), for all *W*_*i*_ ∈ *W*. Then, the expected utilities are *U*_*M*_ = *MB*_*R*_ − *p*_*V*_ *MC*_*V*_ − *nMC*_*A*_ and *U*_*W*_*i*__ = ∣*W*_*i*_∣*WB*_*A*_ − *WC*_*T*_, for each *W*_*i*_ ∈ *W*. In order to maximize the master utility we would like to design games where *p*_*V*_ is small. Therefore, we look for a lower bound on *p*_*V*_. It can be seen using calculus that the largest lower bound is given by the group of minimum size. Although at a first glance this fact seems counterintuitive, it is not surprising due to the following two reasons. On one hand, colluders are likely to be in the majority, but the unique equilibrium occurs when all workers are honest. On the other hand, the extra benefit that workers obtain by colluding is not against the master interest since it is just a saving in computation costs.

## Algorithmic Mechanisms

In this section two realistic scenarios in which the master-worker model considered could be naturally applicable are proposed. For these scenarios, we determine appropriate parameters to be used by the master to obtain the correct answer and maximize its benefit.

The basic protocol (mechanism) used by the master is as follows: Given the payoff parameters (these can either be fixed by the system or be chosen by the master), the master sends to the workers the task to be computed and informs the probability of verification *p*_*V*_. For computational reasons, the master also provides a certificate to the workers. This certificate includes the strategy that the workers must play to achieve the unique NE, together with the appropriate data to demonstrate this fact. (The certificate is included only for cases where resource limitations preclude the worker from computing the unique equilibrium, but it is not related to distributions over public signals as in a correlated equilibrium, since workers do not randomize their choice according to this certificate.)

After receiving the replies from all workers, and independently of the distribution of the answers, the master processor chooses to verify the answers with probability *p*_*V*_. If the answers were not verified it accepts the result of the majority, and it rewards only those workers, whereas if the answers were verified it rewards the correct workers and penalizes the cheaters. The protocol is detailed in Table 3.

**Table 3:** Master algorithm.

1:	**Procedure**
2:	send (task, *p*_*V*_, certificate) to all workers
3:	**upon** receiving all answers **do**
4:	verify the answers with probability *p*_*V*_
5:	**if** the answers were not verified **then**
6:	accept the majority
7:	**end if**
8:	reward/penalize accordingly
9:	**end upon**
10:	**end procedure**

The master, given the payoff parameters, can determine the other parameters, including the value of *p*_*V*_, to force the workers into a unique NE, that would yield the correct task result while maximizing the master’s benefit. Examples of specific parameters such that the master can achieve this are analyzed next.

### SETI-like scenario

The first scenario considered is a volunteer computing system such as SETI@home, where users accept to donate part of their processors idle time to collaborate in the computation of large tasks. In this case, we assume that workers incur in no cost to perform the task, but they obtain a benefit by being recognized as having performed it (possibly in the form of prestige, e.g, by being included on SETI’s top contributors list). In this context, we assume that *WB*_*A*_ > *WC*_*T*_ = 0. The master incurs in a (possibly small) cost *MC*_*A*_ when rewarding a worker (e.g., by advertising its participation in the project). As assumed in the general model, in this model the master may verify the values returned by the workers, at a cost *MC*_*V*_ > 0. We also assume that the master obtains a benefit *MB*_*R*_ > *MC*_*A*_ if it accepts the correct result of the task, and suffers a cost *MP*_*W*_ > *MC*_*V*_ if it accepts an incorrect value.

Under these constraints, replacing in the equations of Section 3, we obtain a unique equilibrium at *p*_*C*_ = 0, that can be enforced making *p*_*V*_ > *WB*_*A*_/(*WP*_*C*_ + 2*WB*_*A*_), attaining **P**_*wrong*_ = 0, *U*_*M*_ = *MB*_*R*_ − *p*_*V*_ *MC*_*V*_ − *nMC*_*A*_, and *U*_*W*_*i*__ = ∣*W*_*i*_∣*WB*_*A*_. Given that in this scenario *p*_*V*_ does not depend on *n*, it is better for the master to allocate the task to minimum number of workers (*n* = 3), maximizing its utility. We highlight this observation in the following theorem.

**Theorem 2**. *For any set of payoff parameters that can be characterized as the SETI scenario, in order to obtain the correct answer (with probability* 1*) while maximizing master’s utility, it is enough to assign the task to only three workers, and verify with probability* *p*_*V*_ > *WB*_*A*_/(*WP*_*C*_ + 2*WB*_*A*_).

### Contractor scenario

The second scenario considered is a company that buys computational power from Internet users and sells it to computation-hungry costumers. In this case the company pays the workers an amount *WB*_*A*_ = *MC*_*A*_ for using their computing capabilities, and charges the consumers another amount *MB*_*R*_ > *MC*_*A*_ for the provided service. Since the workers are not volunteers in this scenario, we assume that computing a task is not free for them (*WC*_*T*_ > 0), and they must have incentives to participate (*U*_*W*_*i*__ > 0, ∀*W*_*i*_ ∈ *W*). The worker payment then must be at least the cost of computing (*WB*_*A*_ ≥ *WC*_*T*_). As in the previous scenario, we assume that the master verifies and has a cost for accepting a wrong value, such that *MP*_*W*_ > *MC*_*V*_ > 0.

Under these constraints, replacing in the equations of Section 3, we obtain a unique equilibrium at *p*_*C*_ = 0, that can be enforced making *p*_*V*_ > (∣*W*_*i*_∣*WB*_*A*_ + *WC*_*T*_)/(∣*W*_*i*_∣(*WP*_*C*_ + 2*WB*_*A*_)), ∀*W*_*i*_ ∈ *W*. Because the latter is decreasing on ∣*W*_*i*_∣, it is enough (a larger lower bound on min_*i*_∣*W*_*i*_∣ could be used if available) to make *p*_*V*_ > (*WB*_*A*_ + *WC*_*T*_)/(*WP*_*C*_ + 2*WB*_*A*_), attaining **P**_*wrong*_ = 0, *U*_*M*_ = *MB*_*R*_ − *p*_*V*_ *MC*_*V*_ − *nMC*_*A*_, and *U*_*W*_*i*__ = ∣*W*_*i*_∣*WB*_*A*_ − *WC*_*T*_.

In this scenario, in addition to choosing the number of workers *n*, we assume that the master can also choose the reward *WB*_*A*_ and the punishment *WP*_*C*_. We focus in the analysis on making only one of these parameters variable at a time. More combinations will be considered in simulations.

***Tunable** n:* The utility of the master *U*_*M*_ = *MB*_*R*_ − *p*_*V*_ *MC*_*V*_ − *nMC*_*A*_ increases as *n* and *p*_*V*_ decrease. Hence, if the number of workers is a choice, using the minimum number of workers (*n* = 3) and the minimum *p*_*V*_ to achieve correctness maximizes the utility. We highlight this observation in the following theorem.

**Theorem 3**. *For any given set of payoff parameters, such that it can be characterized as the contractor scenario, if the master gets to choose the number of workers, in order to obtain the correct answer (with probability* 1*) while maximizing the utility of the master, it is enough to verify with probability* *p*_*V*_ = (*WB*_*A*_ + *WC*_*T*_)/(*WP*_*C*_ + 2*WB*_*A*_) + *ϵ*, *for arbitrarily small* *ϵ* > 0, *assign the task to only three workers*.

***Tunable** WP_C_:* Consider the master utility *U*_*M*_ = *MB*_*R*_ − *p*_*V*_ *MC*_*V*_ − *nMC*_*A*_. That is, *U*_*M*_ = *MB*_*R*_ − (((*WB*_*A*_ + *WC*_*T*_)/(*WP*_*C*_ + 2*WB*_*A*_)) + *ϵ*) *MC*_*V*_ − *nMC*_*A*_. It can be seen that the master utility increases with *WP*_*C*_. However, a large *WP*_*C*_ may discourage workers to join the computation. On the other hand, a large *WP*_*C*_ may alleviate the impact of a large cost of verification *MC*_*V*_. A trade-off between these factors must be taken into account in practice. Nevertheless, this is independent of achieving the equilibrium, as long as the appropriate *p*_*V*_ is used. We highlight these observations in the following theorem.

**Theorem 4**. *For any given sets of workers and payoff parameters, except for* *WP*_*C*_ *that is chosen by the master. If the set of payoffs is such that* *MC*_*A*_ = *WB*_*A*_ > *WC*_*T*_ *and it can be characterized as the contractor scenario, in order to obtain the correct answer (with probability* 1*) while maximizing the utility of the master, it is enough to set* *WP*_*C*_ *as large as possible, verify with probability* *p*_*V*_ = (*WB*_*A*_ + *WC*_*T*_)/(*WP*_*C*_ + 2*WB*_*A*_) + *ϵ*, *for arbitrarily small* *ϵ* > 0.

***Tunable** WB_A_:* Using calculus, it can be seen that, if *WP*_*C*_ > 2*WC*_*T*_ (resp. 2*WC*_*T*_ > *WP*_*C*_), *U*_*M*_ is decreasing (resp. increasing) on *WB*_*A*_. And if on the other hand *WP*_*C*_ = 2*WC*_*T*_, *U*_*M*_ is constant with respect to *WB*_*A*_. For the parameter combinations that make the master utility not constant, because *WB*_*A*_ is limited as *WC*_*T*_ ≤ *WB*_*A*_ < *MB*_*R*_, we have that if *WP*_*C*_ > 2*WC*_*T*_ (resp. 2*WC*_*T*_ > *WP*_*C*_), the utility is maximized when *WB*_*A*_ = *WC*_*T*_ (resp. *WB*_*A*_ = *MB*_*R*_ − *ϵ*, for *ϵ* > 0). Again, this is independent of achieving the equilibrium, as long as the appropriate *p*_*V*_ is used. We highlight these observations in the following theorem.

**Theorem 5**. *For any given sets of workers and payoff parameters, except for* *WB*_*A*_ = *MC*_*A*_ *that is chosen by the master. If the set of payoffs can be characterized as the contractor scenario, in order to obtain the correct answer (with probability* 1*) while maximizing the utility of the master, it is enough to set* *WB*_*A*_ = *WC*_*T*_ *if* *WP*_*C*_ > 2*WC*_*T*_ *or* *WB*_*A*_ = *MB*_*R*_ − *ϵ*_1_ *otherwise, for arbitrarily small* *ϵ*_1_ > 0, *verify with probability* *p*_*V*_ = (*WB*_*A*_ + *WC*_*T*_)/(*WP*_*C*_ + 2*WB*_*A*_) + *ϵ*_2_, *for arbitrarily small* *ϵ*_2_ > 0.

## Simulations

### Simulations design

As shown in Section 3, in order to guarantee a unique equilibrium for any parameter values, all groups must decide whether to cheat or not deterministically (Lemma 1). The reason is algebraic. (Refer to Eq. 3.) If the payoff differentials $\Delta w_{C}^{\left(i\right)}\geq \Delta w_{X}^{\left(i\right)}\geq \Delta w_{\bar{C}}^{\left(i\right)}$ are all positive (resp. negative) the worker-utility differential Δ*U*_*i*_(*s*) is positive (resp. negative) and all workers cheat (resp. are honest) deterministically. But if not all three differentials have the same sign, there could be many values of *p*_*C*_ that make Δ*U*_*i*_(*s*) = 0. That is, there could be multiple mixed equilibria. Since $\Delta w_{C}^{\left(i\right)}\geq \Delta w_{X}^{\left(i\right)}\geq \Delta w_{\bar{C}}^{\left(i\right)}$, to avoid having the same sign in all three differentials, it is enough to make $\Delta w_{C}^{\left(i\right)}>0$ and $\Delta w_{\bar{C}}^{\left(i\right)}<0$ for each group *W*_*i*_ ∈ *W*. That is, from Eqs. 4 and 6, obtain a *p*_*V*_ that satisfies (*WC*_*T*_ − ∣*W*_*i*_∣*WB*_*A*_)/(∣*W*_*i*_∣*WP*_*C*_) < *p*_*V*_ < (∣*W*_*i*_∣*WB*_*A*_ + *WC*_*T*_)/(∣*W*_*i*_∣(*WP*_*C*_ + 2*WB*_*A*_)). And given that ∣*W*_*i*_∣ ≥ 1 and *p*_*V*_ ≥ 0, we get

$$p_{V}<\min_{W_{i}\in W}\frac{|W_{i}|{\mathrm{WB}}_{A}+{\mathrm{WC}}_{T}}{|W_{i}|\left({\mathrm{WP}}_{C}+2{\mathrm{WB}}_{A}\right)}.$$ (7)

Then, plugging the payoff differentials from Eqs. 4 to 6 in Eq. 3, up to *n* − 1 roots may result from making Δ*U*_*i*_ = 0.

Aiming for a mixed equilibrium is promising because it might yield a cost reduction for the master on verifications. However, these multiple equilibria cannot be computed without the knowledge of *all* group sizes, because the computation of the probability of each payoff differential takes into account that each group tosses only one coin (see Eq. 3). Given that only the members of a colluding group know their group size, the computation is not feasible. In fact, although the master is aware of the possible presence of colluders, and the mechanism must take measures against this deviated behavior, the expected worker behavior is not to collude. Hence, the master only has to compute the equilibria for a platform without collusion, and provide these results to workers. That is, fix a *p*_*V*_ restricted to Eq. (7) for ∣*W*_*i*_∣ = 1, and compute the equilibria as the roots of the following polynomial.

ΔwC∑j=0⌊n/2⌋-1n-1jpCn-1-j(1-pC)j+ΔwXn-1⌊n/2⌋pC⌊n/2⌋(1-pC)⌊n/2⌋+ΔwC¯∑j=⌈n/2⌉n-1n-1jpCn-1-j(1-pC)j=0.

Where

$$\begin{array}{cc} \Delta w_{C} & =-p_{V}\;(WP_{C}+2WB_{A})+WB_{A}+WC_{T},\\ \Delta w_{X} & =-p_{V}\;(WP_{C}+WB_{A})+WC_{T},\text{and}\\ \Delta w_{\bar{C}} & =-p_{V}\;WP_{C}-WB_{A}+WC_{T}. \end{array}$$

Then, the colluding groups may use these equilibria, but still take advantage of collusion in the voting outcome, and by sharing the cost of computing when they are honest. We call this the ***compliant behavior***.

Knowing that they will act together, each colluding group could still ignore the master-provided equilibria and decide their own game strategy taking into account their group size. The members of a group do not know the size of other groups, or even their existence. Hence, they can assume nothing but that no worker outside the group colludes. Using this information, the colluding group could compute Nash equilibria, but they cannot enforce the use of such equilibria to other workers. So, they are left only with the choice of using the master-provided equilibria, or drop the aim of achieving equilibrium at all. For instance, a group of colluders may just decide whether to cheat or not deterministically to maximize their expected utility, under the assumption that all other workers are compliant. We call this the ***disobedient behavior***. This scenario can be seen as further deviation (with respect to compliant but voting together) from the expected behavior. The utility differential Δ*U*_*i*_(*s*) (Eq. 3) for a group *W*_*i*_ would be the following.

ΔwC(i)∑j=0⌊n/2⌋-∣Wi∣n-∣Wi∣jpCn-∣Wi∣-j(1-pC)j+ΔwX(i)∑j=⌈n/2⌉-∣Wi∣⌊n/2⌋n-∣Wi∣jpCn-∣Wi∣-j(1-pC)j+ΔwC¯(i)∑j=⌈n/2⌉n-∣Wi∣n-∣Wi∣jpCn-∣Wi∣-j(1-pC)j.

Where $\Delta w_{C}^{\left(i\right)},\Delta w_{X}^{\left(i\right)}$, and $\Delta w_{\bar{C}}^{\left(i\right)}$ are as defined in Eqs. 4 to 6, and *p*_*C*_ corresponds to the equilibria computed by the master (more on how to choose, if many values for *p*_*C*_ are possible, later). Recall that $\Delta U_{i}(s)=U_{i}(s_{-i},s_{i}=C)-U_{i}(s_{-i},s_{i}=\bar{C})$. Then, a colluding group decides to cheat (resp. be honest) if this utility differential is positive (resp. negative).

Finally, it would be interesting to study the impact of having each colluding group choosing an arbitrary *p*_*C*_ at random from (0, 1). We call this the ***random behavior***.

We carry out simulations for all three behaviors defined, setting up *p*_*V*_ so that the game may have multiple mixed equilibria. For the sake of contrast, we also carry out simulations setting up *p*_*V*_ as in the analysis to have a unique pure equilibrium in *p*_*C*_ = 0. The parameter combinations used are shown in Table 4. We set *MB*_*R*_ = *MP*_*W*_ = *MC*_*V*_ = 100, *MC*_*A*_ = *WB*_*A*_, and *WC*_*T*_ = 0.1. For each triplet *n* ∈ {3, 9, 27}, *WB*_*A*_ ∈ [0.1, 2], *WP*_*C*_ ∈ [0, 20], we compute values of *p*_*V*_ that yield mixed and pure equilibria. Then, we proceed as follows.

**Table 4:** Simulation parameters.

behavior	***p*_*C*_** colluder	***p*_*C*_** non-colluder	***p*_*V*_**
pure	0	0	$>\frac{nWB_{A}+WC_{T}}{n(2WB_{A}+WP_{C})}$
compliant	unif. at random from roots of Δ*U*_*i*_(*s*) = 0 in (0, 1)	unif. at random from roots of Δ*U*_*i*_(*s*) = 0 in (0, 1)	$<\frac{WB_{A}+WC_{T}}{2WB_{A}+WP_{C}}$
disobedient	{0, 1}	unif. at random from roots of Δ*U*_*i*_(*s*) = 0 in (0, 1)	$<\frac{WB_{A}+WC_{T}}{2WB_{A}+WP_{C}}$
random	unif. at random from (0, 1)	unif. at random from roots of Δ*U*_*i*_(*s*) = 0 in (0, 1)	$<\frac{WB_{A}+WC_{T}}{2WB_{A}+WP_{C}}$

*MB*_*R*_ = *MP*_*W*_ = *MC*_*V*_ = 100, *MC*_*A*_ = *WB*_*A*_. *n* ∈ {3, 9, 27}, *WB*_*A*_ ∈ [0.1, 2], *WP*_*C*_ ∈ [0, 20], *WC*_*T*_ = 0.1. ⌈*n*/2⌉ groups of size 1 and ⌊*n*/2⌋ of size chosen uniformly at random from [1, ⌊*n*/2⌋].

#### Compliant behavior

We compute the payoff differentials and the roots of the worker-utility differential polynomial. We ignore the roots that are not real numbers in (0, 1). Each of the remaining roots provides a value for *p*_*C*_ that makes the worker-utility differential zero. That is, it is a mixed equilibrium. We then define the group sizes. Given that in practice it is not expected to have a majority of colluders, we fix ⌈*n*/2⌉ workers to be non-colluders, and we choose the remaining group sizes at random in [1, ⌊*n*/2⌋]. Armed with the set of possible *p*_*C*_ values, call it *P*, each group *W*_*i*_ chooses a *p*_*C*_ ∈ *P* uniformly at random. Then, *(i)* we simulate the computation tossing a biased coin for each group according to the *p*_*C*_ value chosen and for the master according to *p*_*V*_, and *(ii)* we compute the expected utility of each worker and the expected utility of the master according to the outcome. Table 5 summarizes these computations.

**Table 5:** Expected utilities.

**verified**	**∣*F*∣**	**master**	**cheater**	**honest**
yes	= *n*	−*MP*_*W*_ − *MC*_*V*_ + *nWP*_*C*_	−*WP*_*C*_	−
yes	< *n*	*MB*_*R*_ − *MC*_*V*_ − (*n* − ∣*F*∣)*MC*_*A*_ + ∣*F*∣*WP*_*C*_	−*WP*_*C*_	*WB*_*A*_ − *WC*_*T*_/∣*W*_*i*_∣
no	> *n*/2	−*MP*_*W*_ − ∣*F*∣*MC*_*A*_	*WB*_*A*_	−*WC*_*T*_/∣*W*_*i*_∣
no	< *n*/2	*MB*_*R*_ − (*n* − ∣*F*∣)*MC*_*A*_	0	*WB*_*A*_ − *WC*_*T*_/∣*W*_*i*_∣

#### Disobedient behavior

For this scenario we proceed in similar fashion for the non-colluding workers. Additionally, for each group, we choose a *p*_*C*_ at random from *P*, and we compute the payoff differentials and the utility differential. The rest of the procedure is the same, except that each colluding group decides deterministically to cheat (resp. be honest) if its utility differential is positive (resp. negative).

#### Random behavior

As before but now each colluding group chooses *p*_*C*_ uniformly at random from (0, 1).

Finally, for the pure equilibrium, we simulate the computation tossing a biased coin only for the master according to *p*_*V*_, given that the workers follow the equilibrium and are honest deterministically. Then, we compute the expected utility of each worker and the expected utility of the master according to the outcome (detailed in Table 5).

Note that we have developed our own simulation platform that integrates Matlab and C++ code to compute roots, simulate coin tossing, and compute utilities for the different behaviors and parameter combinations.

### Discussion and conclusions

Our simulations show that, for the combinations of parameters studied and assuming that the colluders are not a majority, collusion does not help the workers. That is, it is in the best interest of the master *and* also the workers, *even* if they collude, to follow the pure equilibrium *p*_*C*_ = 0. In what follows, we support this observation analyzing plots of the results obtained for *n* = 9. Each dot in these plots represents one execution for one parameter-combination. For many parameter combinations, multiple executions have been carried out obtaining similar results. Similar plots were obtained for *n* = 3 and 27 (included in Supporting Information). Other values of *n* were also simulated obtaining similar results.

**Fig 1:**
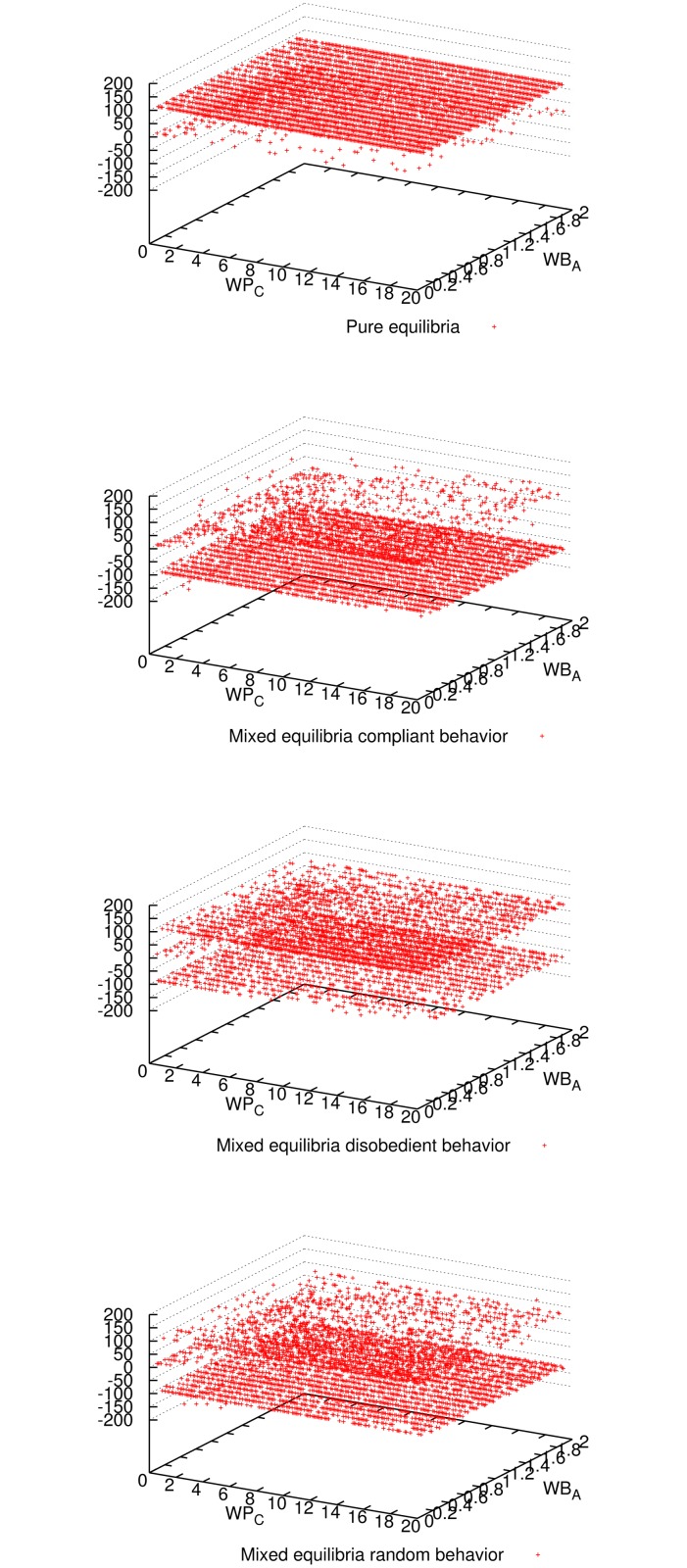
Expected utility of the master for all three mixed-equilibria behaviors and the pure equilibrium and for different parameter combinations when 9 workers participate. It can be seen that enforcing a pure equilibrium is the best for the master utility for most of the parameter combinations.

**Fig 2:**
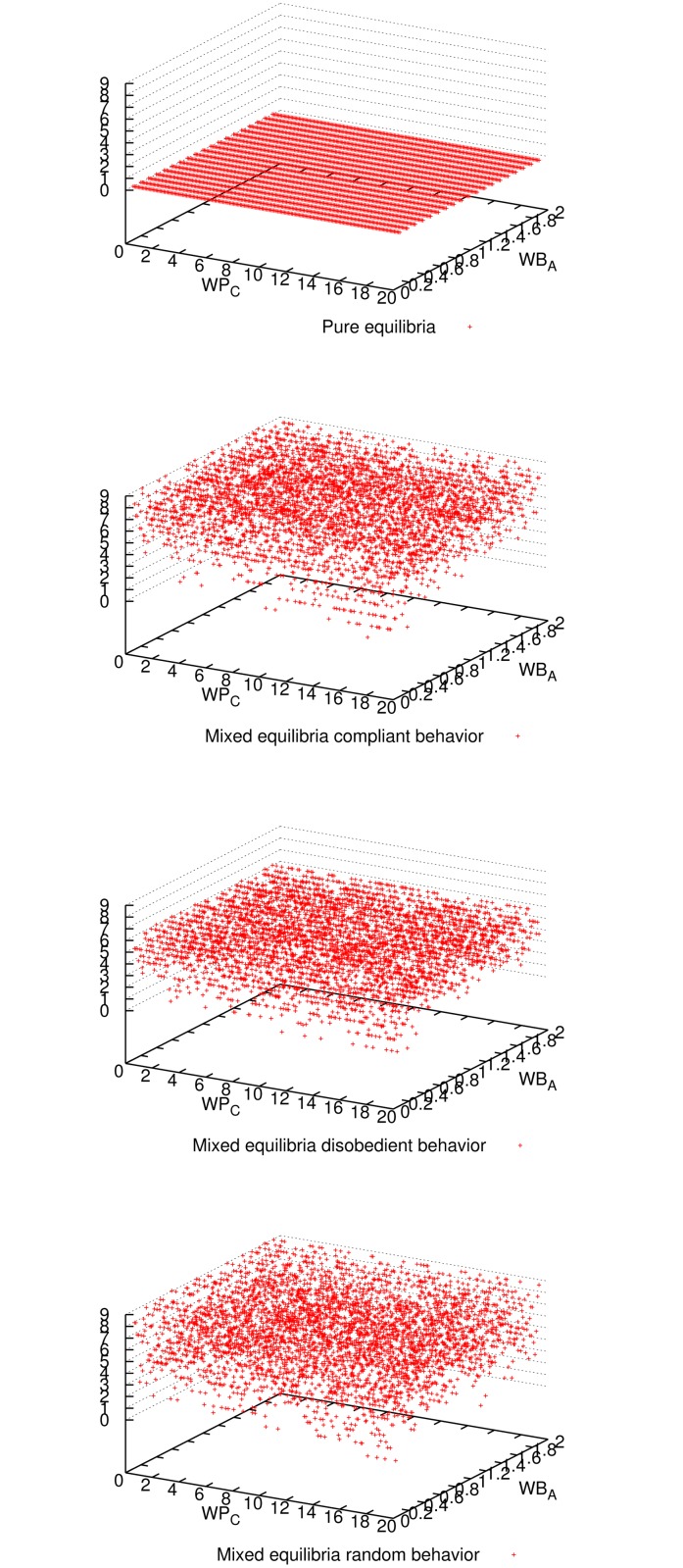
Number of cheaters for all three mixed-equilibria behaviors and the pure equilibrium and for different parameter combinations when 9 workers participate. As expected, enforcing the pure equilibrium (*p*_*C*_ = 0) workers are honest for all parameter combinations.

**Fig 3:**
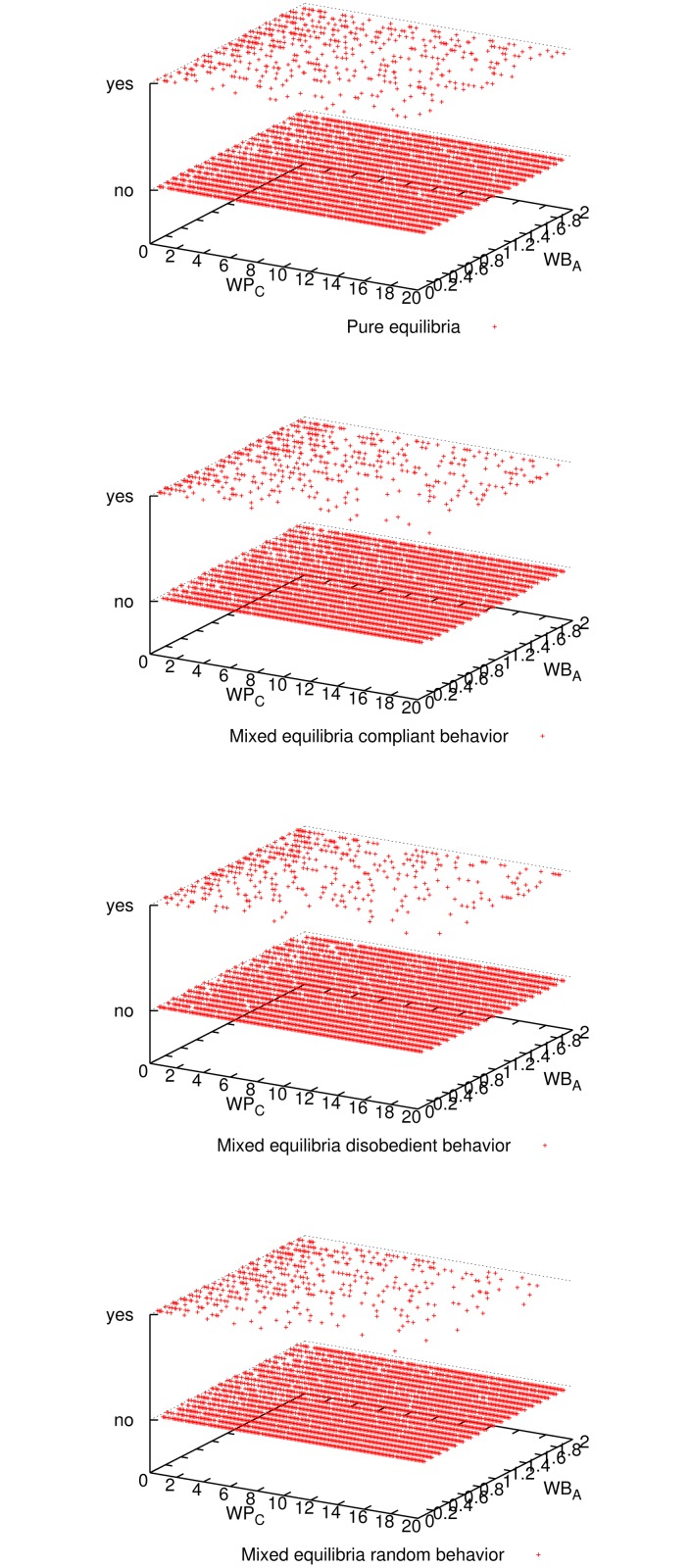
Outcome of master’s decision (verify or not) for all three mixed-equilibria behaviors and the pure equilibrium and for different parameter combinations when 9 workers participate. Given the low probability of verification needed, the master did not verify for most of the parameter combinations in all four cases.

**Fig 4:**
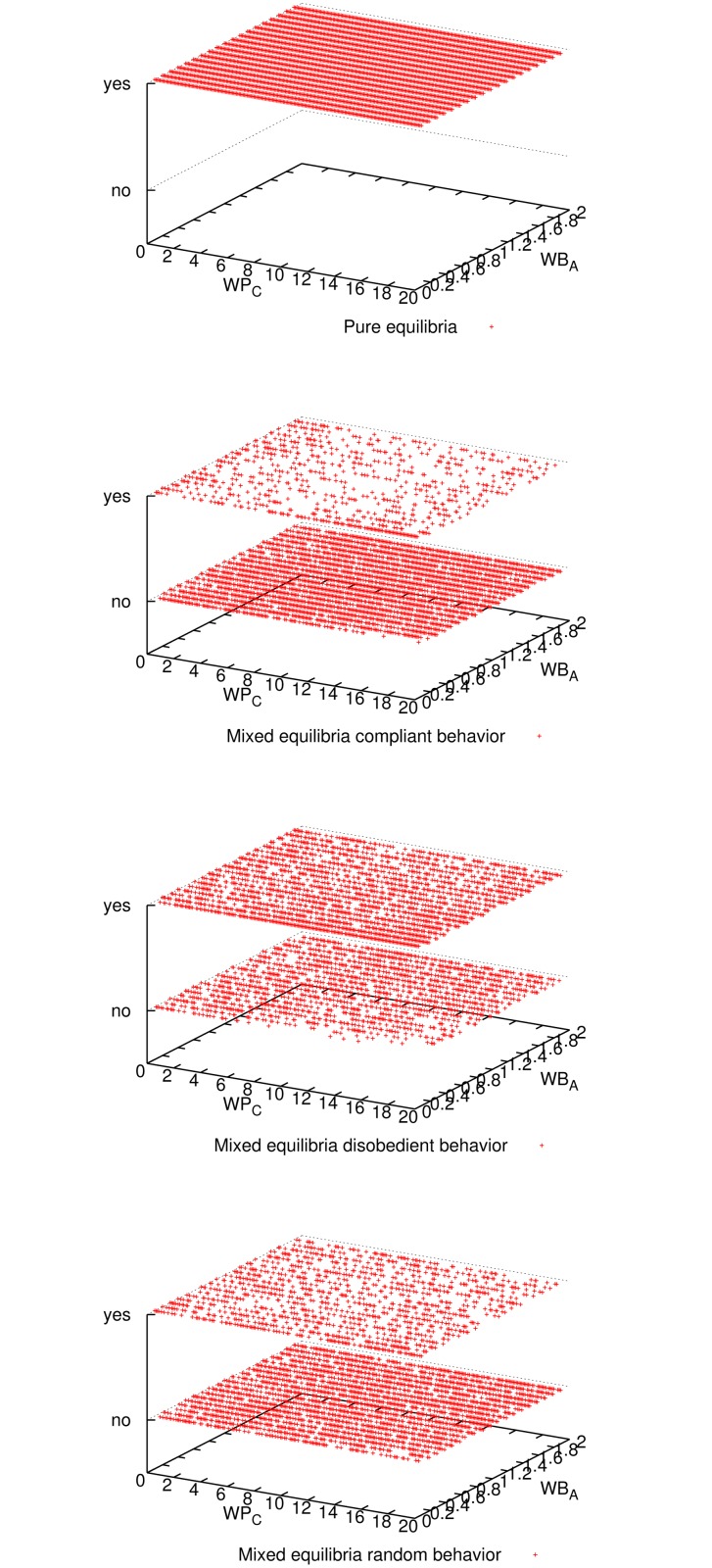
Outcome of the computation (correct or incorrect result) for all three mixed-equilibria behaviors and the pure equilibrium and for different parameter combinations when 9 workers participate. It can be seen that aiming for a pure equilibrium (*p*_*C*_ = 0) is the only option to guarantee correctness for any parameter combination.

**Fig 5:**
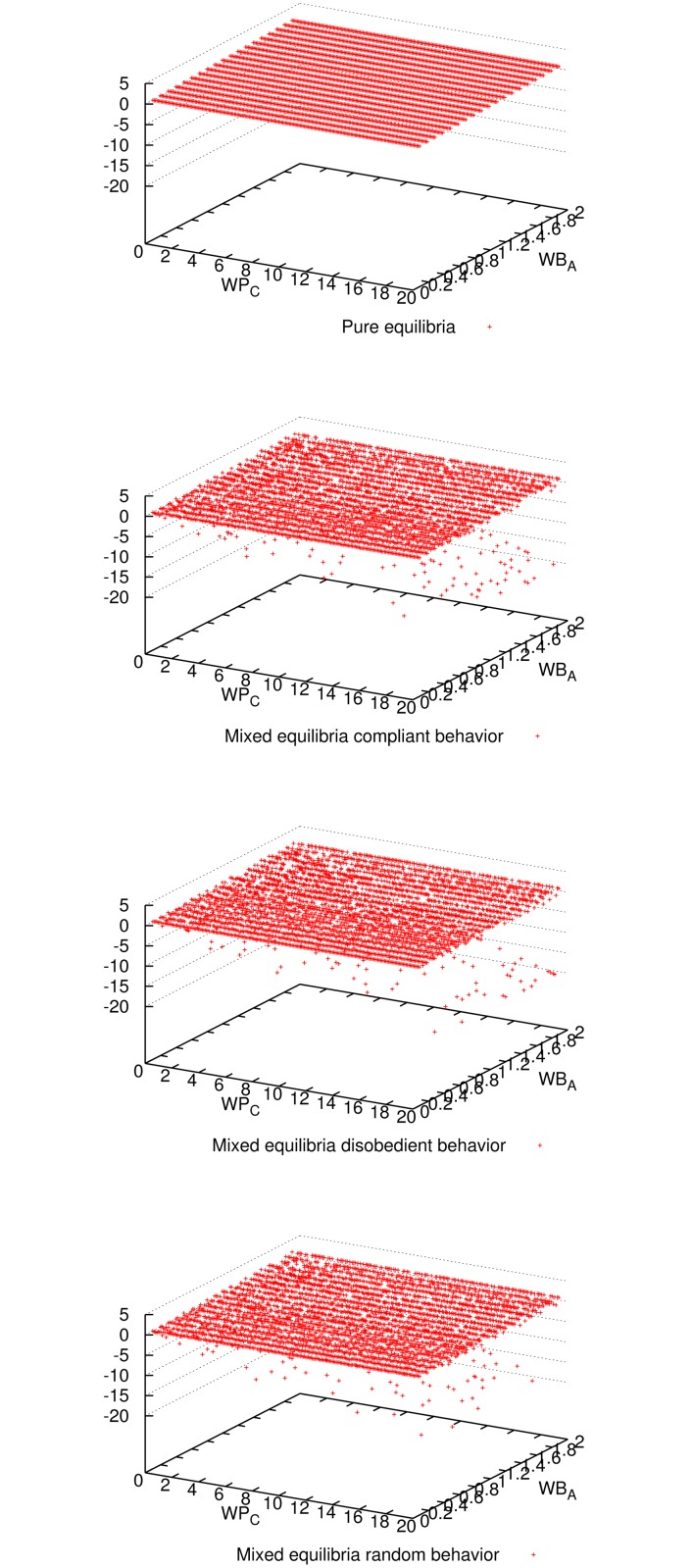
Expected utility of non-colluder workers for all three mixed-equilibria behaviors and the pure equilibrium and for different parameter combinations, when 9 workers participate. It can be seen that aiming for a pure equilibrium is the best for the non-colluder utility for most of the parameter combinations.

**Fig 6:**
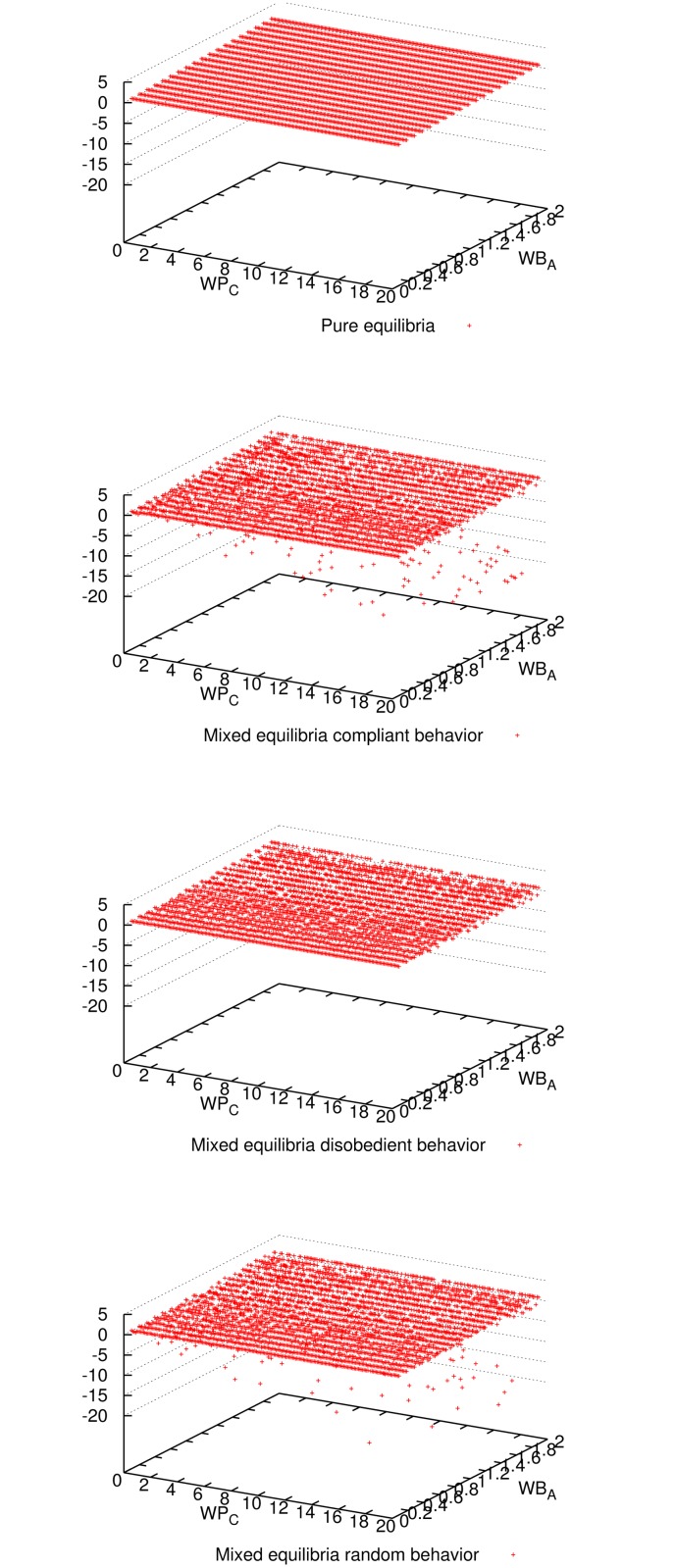
Expected utility of colluder workers for all three mixed-equilibria behaviors and the pure equilibrium and for different parameter combinations when 9 workers participate. It can be seen that aiming for a pure equilibrium is the best for the colluder utility for most of the parameter combinations.

**Fig 7:**
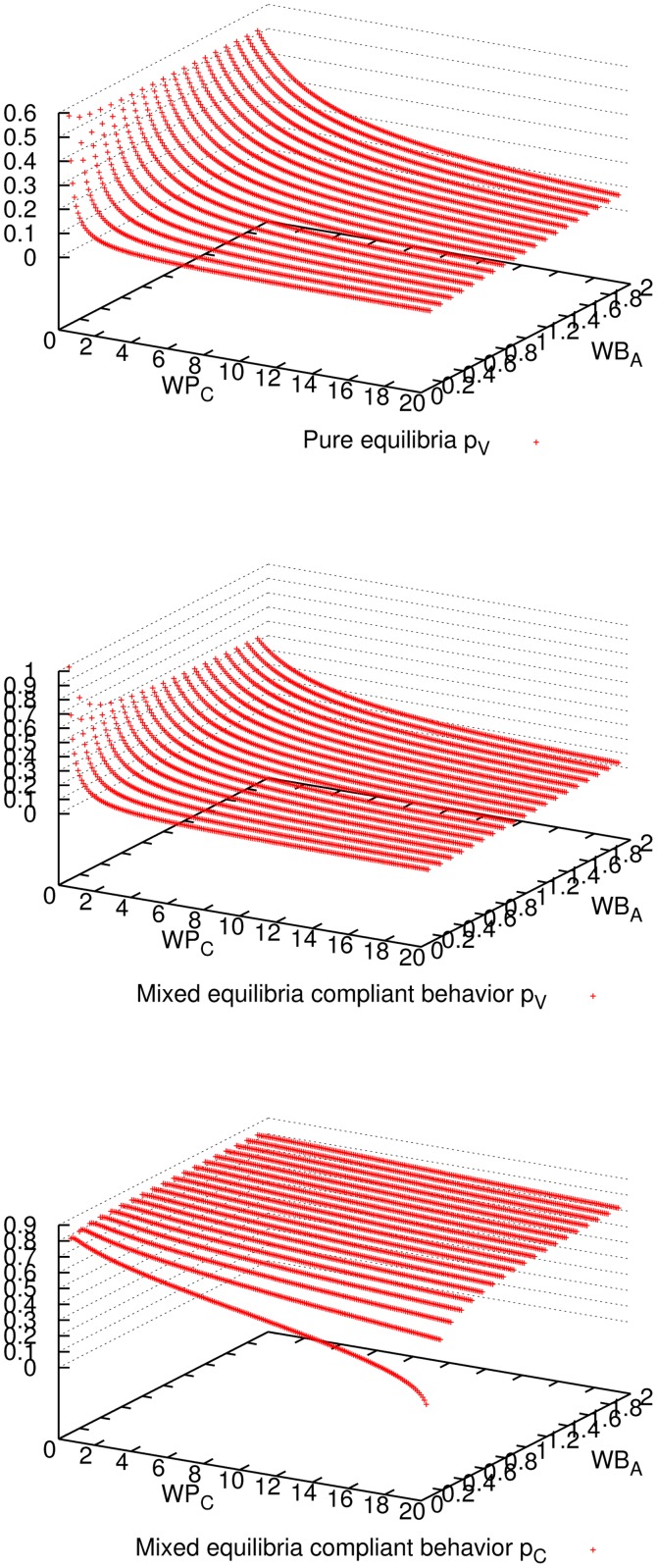
Probability of verification of the master for pure equilibrium and mixed equilibria, and probability of cheating of a compliant non-colluder worker, for different parameter combinations when 9 workers participate. For the master, it can be seen that, for most of the parameter combinations, the probability of verifying is very low.

Fig. 1 shows the expected utility of the master for all three mixed-equilibria behaviors and the pure equilibrium. Comparing all four cases, we observe that enforcing a pure equilibrium is the best for the master expected utility, even though it has to reward all workers because they never cheat (cf. Fig. 2). The master verifies more frequently for the pure equilibrium (cf. Fig. 3), but still not so frequently to impact in the expected utility (refer to Fig. 1), and on the other hand it is always correct (see Fig. 4). Having a platform where the master is always correct and its expected utility is maximized, the natural question is how good is the situation for workers. It can be seen in Fig. 5 and 6 that the expected utility of workers is also higher if the pure equilibrium is used. And this is the case for the non-colluders (Fig. 5) as well as for the colluders (Fig. 6). Finally, we also observed that the probability of verifying *p*_*V*_ and the *p*_*C*_ computed by the master are both significantly smaller if the ratio penalty/payoff is large as one might expect (see Fig. 7).

## Supporting Information

S1 Fig Simulation plots for 3 workers. The same observations for the case of 9 workers in Figs. 1 to 7 apply to this case.
(EPS)

S2 Fig Simulation plots for 27 workers. The same observations for the case of 9 workers in Figs. 1 to 7 apply to this case.
(EPS)

## References

[1] Korpela E, Werthimer D, Anderson D, Cobb J, Lebofsky M (2001) SETI@home: Massively distributed computing for SETI. Computing in Science and Engineering 3(1):78–83.

[2] Anderson D (2004) BOINC: A system for public-resource computing and storage. Proceedings of the 5th IEEE/ACM International Workshop on Grid Computing 4–10. 10.1109/GRID.2004.14

[3] Amazon’s Mechanical Turk. Available: https://www.mturk.com. Accessed 13 November 2014.

[4] Golle P, Mironov I (2001) Uncheatable distributed computations. Proceedings of the 2001 Conference on Topics in Cryptology: The Cryptographer’s Track at RSA 425–440. 10.1007/3-540-45353-9_31

[5] Anderson D (2010) Volunteer computing: the ultimate cloud. Crossroads 16(3):7–10. 10.1145/1734160.1734164

[6] Kondo D, Araujo F, Malecot P, Domingues P, Silva L, Fedak G, Cappello F (2007) Characterizing result errors in Internet desktop grids. Proceedings of the 13th International Euro-Par Conference European Conference on Parallel and Distributed Computing 361–371.

[7] Fernández Anta A, Georgiou Ch, Lopez L, Santos A (2012) Reliable Internet-based computing in the presence of malicious workers. Parallel Processing Letters 22(1):1250002 10.1142/S0129626412500028

[8] Konwar KM, Rajasekaran S, Shvartsman AA (2006) Robust network supercomputing with malicious processes. Proceedings of the 20th International Symposium on DIStributed Computing 474–488.

[9] Sarmenta L (2002) Sabotage-tolerance mechanisms for volunteer computing systems. Future Generation Computer Systems 18(4):561–572. 10.1016/S0167-739X(01)00077-2

[10] Abraham I, Dolev D, Goden R, Halpern JY (2006) Distributed computing meets game theory: Robust mechanisms for rational secret sharing and multiparty computation. Proceedings of the 25th Annual ACM SIGACT-SIGOPS Symposium on Principles of Distributed Computing 53–62.

[11] Shneidman J, Parkes DC (2003) Rationality and self-interest in P2P networks. Proceedings of the 2nd International Workshop on Peer-to-Peer Systems 139–148. 10.1007/978-3-540-45172-3_13

[12] Christoforou E, Fernández Anta A, Georgiou Ch, Mosteiro MA, Sánchez A (2013) Applying the dynamics of evolution to achieve reliability in master-worker computing. Concurrency and Computation: Practice and Experience 25(17):2363–2380. 10.1002/cpe.3104

[13] Fernández Anta A, Georgiou Ch, Mosteiro MA (2008) Designing mechanisms for reliable Internet-based computing. Proceedings of the 7th IEEE International Symposium on Network Computing and Applications 315–324.

[14] Yurkewych M, Levine BN, Rosenberg AL (2005) On the cost-ineffectiveness of redundancy in commercial P2P computing. Proceedings of the 12th ACM Conference on Computer and Communications Security 280–288.

[15] Christoforou E, Fernández Anta A, Georgiou Ch, Mosteiro MA (2014) Algorithmic mechanism design for reliable master-worker Internet-based computing. IEEE Transactions on Computers 63(1):179–195. 10.1109/TC.2012.186

[16] Christoforou E, Fernández Anta A, Georgiou Ch, Mosteiro MA, Sánchez A (2013) Reputation-based mechanisms for evolutionary master-worker computing. Proceedings of the 17th International Conference on Principles of Distributed Systems 98–113 10.1007/978-3-319-03850-6_8

[17] Nisan N, Roughgarden T, Tardos T, Vazirani VV, editors (2007) Algorithmic Game Theory. Cambridge University Press 778 p.

[18] Abraham I, Alvisi L, Halpern JY (2011) Distributed computing meets game theory: combining insights from two fields. ACM SIGACT News 42(2):69–76. 10.1145/1998037.1998055

[19] Cosmin G, Araujo F, Moura Silva L, Domingues P, Arenas AE (2009) Defeating colluding nodes in desktop grid computing platforms. Journal of Grid Computing 7(4):555–573. 10.1007/s10723-009-9124-5

[20] Staab E, Engel T (2009) Collusion Detection for Grid Computing. Proceedings of the 9th IEEE/ACM International Symposium on Cluster Computing and the Grid 412–419.

[21] Kondo D, Casanova H, Wing E, Berman F (2002) Models and scheduling mechanisms for global computing applications. Proceedings of the 16th IEEE International Parallel and Distributed Processing Symposium 79–86

[22] Douceur JR (2002) The Sybil attack. Proceedings of the 1st International Workshop on Peer-to-Peer Systems 251–260 10.1007/3-540-45748-8_24

[23] Ding S, Xia CY, Zhou KL, Yang SL, Shang JS (2014) Decision support for personalized cloud service selection through multi-attribute trustworthiness evaluation. PLoS ONE 9(6) e97762 10.1371/journal.pone.0097762 24972237 PMC4074036

[24] Ding S, Yang SL, Zhang YT, Liang CY, Xia CY (2014) Combining QoS prediction and customer satisfaction estimation to solve cloud service trustworthiness evaluation problems. Knowledge-Based Systems 56:216–115. 10.1016/j.knosys.2013.11.014

[25] Nisan N, Ronen A (2001) Algorithmic mechanism design. Games and Economic Behavior 35:166’196. 10.1006/game.1999.0790

[26] Nash JF (1950) Equilibrium points in *n*-person games. Proceedings of the National Academy of Sciences 36(1):48–49. 10.1073/pnas.36.1.48 PMC1063129 16588946

[27] Babaioff M, Feldman M, Nisan N (2006) Combinatorial agency. Proceedings of the 7th ACM Conference on Electronic Commerce 18–28.

[28] Taufer M, Anderson D, Cicotti P, Brooks CL (2005) Homogeneous redundancy: a technique to ensure integrity of molecular simulation results using public computing. Proceedings of the 19th IEEE International Parallel and Distributed Processing Symposium 119a 10.1109/IPDPS.2005.247

[29] Golle P, Stubblebine S (2001) Secure distributed computing in a commercial environment. Proceedings of the 5th International Conference Financial Cryptography 289–304.

[30] Osborne MJ (2003) An Introduction to Game Theory. Oxford University Press 560 p.

